# Reviews

**Published:** 1908-05-02

**Authors:** 


### REVIEWS.

Burdett's Hospitals and Charities, 1908 : The Year-book of Philanthropy and the Hospital Annual. By Sir Henry Burdett, K.C.B. (London : The Scientific Press, Ltd.). Pp. 932. Price, 7s. 6d. net.

The new edition of "Burdett's Hospitals and Charities" retains all the useful characteristics which are familiar to those interested in the practical side of charity. As an exhaustive reference book this volume continues to stand alone. The large mass of statistical information, dealing with the administration and finance of hospitals and other benevolent institutions throughout the English-speaking world, will be found as full and as clearly arranged as in previous years. Particulars of the chief nursing institutions and associations throughout the country, and of the more important continental nursing homes, have been re-inserted. Another useful addition to this year's issue is the telephone numbers of the hospitals of London. The early chapters, which deal with the wider aspects of organised philanthropy, are more than usually interesting, and deserve careful attention. That upon "The Volume of Charity" disposes once again of the contention that there is a fixed yearly maximum contribution which can be exacted from the public for the purposes of charity. This edition clearly shows that what was demonstrated in its pages ten years ago is equally true of to-day. The possible volume of charity is not a fixed sum ; indeed there has been a remarkable increase during the past decade. Another point which should be noted is the comparison which is drawn between the financial progress of foreign missions and that of the Bishop of London's Fund.

